# Reasoning with the inverse of 3D cardinal direction relations based on direction matrices

**DOI:** 10.1038/s41598-024-66130-w

**Published:** 2024-07-05

**Authors:** Miao Wang, Xingxing Dong, Jixun Gao, Zhenxi Fang, Hao Tang, Song Li

**Affiliations:** 1 Department of Software, Henan University of Engineering, Zhengzhou, 451191 China; 2 Department of Computer Science and Technology, Henan Polytechnic University, Jiaozuo, 454000 China; 3 Department of Computer Science, Zhongyuan University of Technology, Zhengzhou, 450007 China; 4 College of Computer Science and Technology, Harbin University of Science and Technology, Harbin, 150080 China

**Keywords:** Solid Earth sciences, Space physics, Engineering, Mathematics and computing

## Abstract

In order to improve the ability of intelligent reasoning and prediction of 3DR27 model which presented in our previous work, enhance the usability of this model, and better fulfill the demands of real applications for spatial database, we focused on the problem of reasoning with the inverse of 3D cardinal direction relations between spatial objects. In order to realize automated reasoning, an algorithm for computing the inverse of 3D cardinal direction relations based on matrices is proposed on the basis of the mapping between the 3D rectangular cardinal direction relations and 3D interval relation matrix. This algorithm improves the power of reasoning for this model by means of the excellent properties of matrix operations. Theorems are provided to prove formally that our algorithm is correct and complete. This study realized the automatic inference and calculation of the inverse of the 3D cardinal direction relations based on 3DR27 model and further improved the ability of spatial reasoning and spatial analysis of spatial database.

## Introduction

Direction relation is an important part of spatial relations, which reflects the order relations between spatial objects, such as front side, back side, left side, right side, etc. It is widely used in many fields such as Spatial Intelligent Analysis and Processing, Urban Pipeline Network, Robot Navigation, Disaster Prevention and Mitigation, etc.^1,2^. And it is increasingly becoming a hot and difficult problem in the fields of data modeling, cartographic generalization, multimedia design, image retrieval, etc.^3^. The representation and reasoning with direction relation is a fundamental problem in the research field of spatial direction relations^4^, which is important in Spatial Retrieval^5,6^, Spatial Localization^7,8^, Quality Inspection^9,10^ and Spatial Storage^11^ in the field of spatial database.

We know that the inverse operation for direction relations is widely used in many real applications. It allows to infer the direction of object *b* with respect to *a*, given the direction of object *a* with respect to *b*, which plays an important role in saving storage and improving the efficiency of spatial queries and spatial reasoning^12,13^. For example, in the intelligent transportation system, the results of the inverse operation with direction relations can be used as the constraints for path selection to reduce the search range and then improve the search efficiency. When traffic congestion and traffic accidents occur, it can provide decision-making and support for selecting reasonable travel paths.

In our work, we aim to solve the problem of automatically computing the inverse of 3D cardinal direction relations in 3DR27 model^14^. This model is currently one of the most expressive models for cardinal directions between 3D space. Because it is simple in calculation and it is easy to perform formal reasoning. The specific contributions of this paper are summarized as follows.

- We introduce the operations of the 3 × 3 × 3 direction relations matrix to study the inversion operation for cardinal direction relations in the model of our previous work. We give the representation of the rectangular cardinal direction relations in the form of interval matrix. We get the results of the inversion operation of interval matrix by using the inference rules of interval algebra.
- Then, we study the equivalent correlations between the 3D interval relation matrix and the 3D rectangular cardinal direction relations. We give several important theorems which provide the solution for the target problem by using the operations of direction matrix, and their proofs to build up our theoretical framework.
- Before the algorithm for computing the inverse of 3D cardinal direction relations based on matrix is presented, we focus on the problem of computing the original relation for basic 3D rectangular cardinal direction relations.
- And we propose an algorithm to compute all possible results of the inverse of the basic 3D cardinal direction relations directly. Several theorems are provided to prove that our algorithms are correct. In our work, we solve the problem of the automatically computing the inverse operation of the 3D cardinal direction relations by means of the excellent properties of matrix operations, after the process of Projection, Inversion, Cartesian product, and solving the original relation.
- Then, we implement our algorithm in C programs. The results of running the programs show that our algorithm is correct and complete. In this paper, we realized the automatic calculation of the inverse of the basic cardinal direction relations in the model of our previous work. Our study makes 3DR27 model has the power of automated reasoning for the inverse operation because our solution does not require the help of reference tables and any manual calculation.

This article is structured as follows. In “Related work” section, we survey related works. In “3D direction relations model” section presents the 3DR27 model. In “3D direction relation matrix” section, we introduce the operations of the 3 × 3 × 3 direction relation matrix to study the inversion operation for cardinal direction relations in the 3DR27 model. And introduce the equivalent correlations between the 3D interval relation matrix and the 3D rectangular cardinal direction relations. In “Computing the inverse of 3D cardinal direction relations based on matrices” section presents our algorithms for the inverse operation of 3D cardinal direction relations and proves the correctness of our algorithm. In “Verification results and discussions” section discusses the implementation of the proposed algorithms and presents the results of analysis and verification. Finally, “Conclusion” section concludes the paper and discusses future work.

## Related work

In recent years, many models for direction relations have been proposed in 2D space, such as cone model^15^, MBR model^16^, direction relations matrix model^17^, and Voronoi diagram model^18^. In the cone model, the spatial objects are approximately modeled as points, which reduces the precision of expression and reasoning. The direction relations model based on MBR determines the direction relations between spatial objects by using the direction relations between the minimum bounding rectangles of the spatial objects, which takes into account the influence of their sizes and shapes of the spatial objects to a certain extent. However, the model does not consider the case where objects intersect each other.

The direction relations matrix model is currently one of the most expressive models for cardinal directions in 2D space because it can more realistically represent the direction relations between regions, and it is simple in calculation and easy to perform formal reasoning. The Voronoi diagram model considers the characteristics of the spatial object by means of the feature points of the spatial object instead of the spatial object, but there is still a gap with the description of the spatial relations for the real objects. Then, many improved models have been proposed. Wang et al.^19^ proposed a position expression model based on qualitative coordinates, which describes the direction relations between the primary object and the reference object by recording the qualitative positional region of the primary object and the reference object, and incorporates the distance relations. The models considers the shape of the spatial object to a certain extent, and improves the accuracy of the model expression and reasoning.

In a previous line of work, Li et al.^20^ improved the similarity calculation model of direction relations proposed by Goyal^17^, which further extends the application of direction relation matrix model. The results of similarity calculation are more accurate, but the model cannot describe the situation where objects intersect each other. Chen et al.^21^ proposed a direction Voronoi diagram model by analyzing the formal description model of spatial direction relations. The model can better reflect the influence of the shape and size of the group targets, but it does not consider the influence of many factors at the same time. Wang^22^ proposed a new model of spatial orientation relationship combining with the idea of fuzzy mathematics, which is more consistent with the orientation cognition habits of people.

Although, many models for direction relations in 3D space have been proposed, the ability of intelligent reasoning for these models is urgent to be enhanced. Pacheco et al.^23,24^ extended the research object to 3D space and proposed the reference object as a point for the first time, which is applicable to simpler spatial objects and has low in accuracy for description. Wang et al.^14^ proposed a direction relation qualitative representation and reasoning model in 3D space, which is the extension of direction relation matrix model on the plane. The model realized a more accurate division of the direction relations and made the description of spatial direction relations simpler, which improved the ability of spatial reasoning, and laid the foundation for further applications. Chen et al.^25^ studied the interaction cube matrix to represent the direction relations of 3D spatial objects, but it cannot cover all possible direction relations. The 3DR39 model adds several direction tiles on the basis of 3DR27 model, but the description accuracy still needs to be further improved.

There are many improved models, such as the direction relations matrix model based on double projections^26^, 3DR39 model^27^, 3DR44-4d model^28^, 3DR34 model^29^, the enhanced 3DR34 model^30^, 3DR44 model^31^, 3DR46 model^32^, and many other models, which are essentially further subdivisions of the space. The 3DR27 model is currently one of the most expressive models for cardinal directions in 3D space because it is simple in calculation and it is easy to perform formal reasoning, and several works on reasoning with direction based on this model has been carried out one after another. We studied the problem of composing 3D cardinal direction relations defined by 3DR27 model presented in^14^ from view of block algebra, which improves the powers of reasoning of the model. However, it did not realize the automatically computing the inverse of 3D cardinal direction relations, and still require the help of reference tables and any manual calculation. Then, with the help of the *n*-dimensional algebra of Balbiani, Wang et al.^33^ proposed an algorithm for computing the composition of the basic 3D cardinal direction relations defined by the 3DR27 model based on block algebra.

In addition, the previous researches^34–37^ mainly focus on computing the inverse of cardinal direction relations in 2D space. However, the real world is a three-dimensional space. The abilities of intelligent reasoning for directions in three-dimensional space is urgent to be enhanced. Wang et al.^38^ studied the inverse operation of basic rectangle cardinal direction relations defined by 3DR27 model by means of 3D algebra theory. By using the equivalent connection between 3D algebra and 3D rectangle cardinal direction relations, the inverse of 3D rectangle cardinal direction relations is transformed into the inverse operation on the equivalent 3D algebra relations. On this basis, we^39^ studied the correlation between 3D rectangular cardinal direction and 3D block algebra relations, and then proposed an algorithm for computing the inverse of the 3D rectangular cardinal direction relations. What is more, we introduce the theory of block algebra into the research area of reasoning with the inverse of the 3D cardinal direction relations. Then, Wang et al.^40^ studied the concept of the original relation of the basic rectangular cardinal direction relations of the 3DR27 model. And with the help of the original relation of the 3D rectangular cardinal direction relations, we proposed the inverse operation of the basic 3D cardinal direction relations in the model, but the method still depend on the help of reference tables of the 3D rectangular cardinal direction relations and manual calculation. In order to avoid the drawbacks of the existing method, Hao et al.^41^ devised a new model that can deal with and analyze a large number of complex directions and positions, and solved the problem of composing the inverse of the direction relations defined by the 3DR39-3d model, but the inference accuracy still needs to be further improved.

Although the current 3D direction relations models gradually refines the division of space, the power of intelligent reasoning for these models is urgent to be enhanced. The spatial reasoning still depends on the help of reference tables or manual calculation. So, there is an urgent need to develop the ability of intelligent reasoning.

In this paper, we propose an algorithm for computing all possible results of the inverse of the 3D cardinal direction relations based on matrix in the 3DR27 model, which realizes the automatic reasoning and calculation of inverse operation for direction relations by means of matrix operation. The algorithm solves the problem of automatically computing the inverse of 3D cardinal direction relations, and further improves the ability of spatial reasoning and spatial analysis of spatial database.

## 3D direction relations model

**Figure 1:**
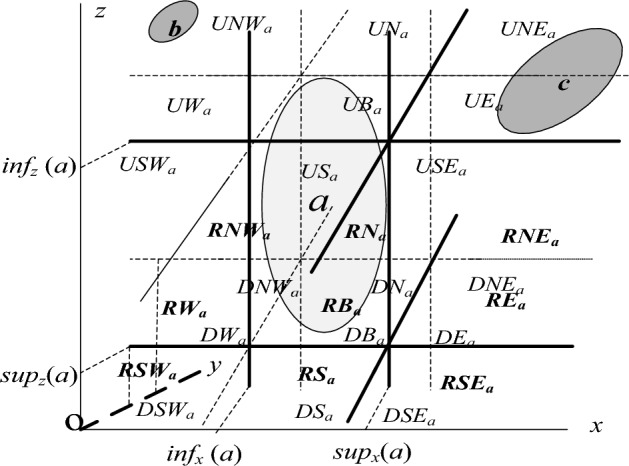
Space division of 3DR27 model.

In this paper, the 3DR27 model presented in our previous work^14^ (see Fig. 1) is used to describe the direction relations between 3D spatial objects. Let us show this model formally through the following definitions. Objects concerned in this study are homeomorphic to the closed box ({(*x*, *y*, *z*): *x*^2^ + *y*^2^ + *z*^2^ $\le$ 1}). The set of these spatial objects is denoted as *Tbox*.

### Definition 1^33^

Let *a* $\in$ *Tbox*. The greatest lower bound of the projection of *a* on the *x*-axis (respectively *y*-axis, *z*-axis) is denoted by *inf*_x_(*a*) (respectively *inf*_*y*_(*a*), *inf*_*z*_(*a*)). The least upper bound or the supremum of the projection of *a* on the *x*-axis (respectively *y*-axis, *z*-axis) is denoted by *sup*_*x*_(*a*) (respectively *sup*_*y*_(*a*) *sup*_*z*_(*a*)). The minimum bounding box of a object *a*, denoted by *mbb*(*a*), is the cube formed by the straight lines *x* = *inf*_*x*_(*a*), *x* = *sup*_*x*_(*a*), *y* = *inf*_*y*_(*a*), *y* = *sup*_*y*_(*a*) *z* = *inf*_*z*_(*a*)and *z* = *sup*_*z*_(*a*).

### Definition 2^33^

Let *a* $\in$ *Tbox* and *a* be reference object. The straight lines *x* = *inf*_*x*_(*a*), *x* = *sup*_*x*_(*a*), *y* = *inf*_*y*_(*a*), *y* = *sup*_*y*_(*a*) *z* = *inf*_*z*_(*a*)and *z* = *sup*_*z*_(*a*) forming *mbb*(*a*) divide the space into 27 areas which we call tiles of *a*. These tiles will be denoted by *UNW*_*a*_, *UN*_*a*_, *UNE*_*a*_, *UW*_*a*_, *UB*_*a*_, *UE*_*a*_, *USW*_*a*_, *US*_*a*_, *USE*_*a*_, *RNW*_*a*_, *RN*_*a*_, *RNE*_*a*_, *RW*_*a*_, *RB*_*a*_, *RE*_*a*_, *RSW*_*a*_, *RS*_*a*_, *RSE*_*a*_, *DNW*_*a*_, *DN*_*a*_, *DNE*_*a*_, *DW*_*a*_, *DB*_*a*_, *DE*_*a*_, *DSW*_*a*_, *DS*_*a*_ and *DSE*_*a*_, respectively.

### Definition 3^14^

Let *a*, *b* $\in$ *Tbox* where *a* is the reference object and *b* is the primary object. If *b* is included in tile *UNW*_*a*_ of *a* then we say that *b* is up-northwest of *a* and we write ***b UNW a***. Similarly, we can define up-north(*UN*), up-south (*US*), up-northeast (*UNE*), up-west (*UW*), up-bounding box (*UB*), up-southwest (*USW*), up-east (*UE*), up-southeast (*USE*), north (*RN*), south (*RS*), northeast (*RNE*), west (*RW*), bounding box (*RB*), southwest (*RSW*), east (*RE*), southeast (*RSE*), northwest (*RNW*), down-north (*DN*), down-south (*DS*), down-northeast (*DNE*), down-west (*DW*), down-bounding box (*DB*), down-southwest (*DSW*), down-east (*DE*), down-southeast (*DSE*) and down- northwest (*DNW*) relations. The above relations called single-tile cardinal direction relations.

### Example 1

As shown in Fig. 1, the primary object *b* is included in the tile *UNW*_*a*_, then we have *b UNW a* holds.

However, the primary object may fall into more than one tile. For instance the primary object *c* lies partly in the tile *UNE*_*a*_ and partly in the tile *UE*_*a*_ of *a* (Fig. 1), then we say that *c* is partly up-northwest and partly up- east of *a* and we write *c UNE*:*UEa*.

Without loss of generality, the definition of basic cardinal direction relation is defined by 3DR27 model on the basis of the single-tile cardinal direction relations as following.

### Definition 4^14^

A basic 3D cardinal direction relations under 3DR27 model is an expression *R*_1_: … :*R*_*k*_ where *R*_1_, …, *R*_*k*_ $\in${*UNW*, *UN*, *UNE*, *UW*, *UB*, *UE*, *USW*, *US*, *USE*, *RNW*,* RN*, *RNE*, *RW*, *RB*, *RE*, *RSW*, *RS*, *RSE*, *DNW*, *DN*, *DNE*, *DW*, *DB*, *DE*, *DSW*, *DS*, *DSE*}, 1 ≤ *k* ≤ 27, and *R*_*i*_* ≠ R*_*j*_ for every *i*, *j* such that 1 ≤ *i*, *j* ≤ *k* and *i ≠ j*, and there exist objects *b*_1_, …, *b*_*k*_ $\in$ *T*_*box*_ such that *b*_1_ $\in$ *R*_1*a*_, …, *b*_*k*_ $\in$ *R*_*ka*_ and *b*_1_ ∪ ··· ∪ *b*_*k*_ $\in$ *T*_*box*_ for any reference object *a* $\in$ *T*_*box*_. The set of basic 3D cardinal direction relations in this model is denoted by *TD*.

The 3DR27 model describes the cardinal direction relation of the primary region* b* relative to the reference region *a* by using a Boolean $3\times 3\times 3$ matrix (Eq. 1) called the direction relation matrix. Such matrix is generated by checking whether the intersection of the primary object and the tiles of the reference object is empty or not. For each tile, if such intersection is non-empty, it means that the primary object falls into this direction tile.

1 $$Dir_{3 \times 3 \times 3} \left( {b,a} \right) = \left[ {\left[ {\begin{array}{*{20}l} {UNW_{a} \cap b} \hfill & {UN_{a} \cap b} \hfill & {UNE_{a} \cap b} \hfill \\ {UW_{a} \cap b} \hfill & {UB_{a} \cap b} \hfill & {UE_{a} \cap b} \hfill \\ {USW_{a} \cap b} \hfill & {US_{a} \cap b} \hfill & {USE_{a} \cap b} \hfill \\ \end{array} } \right]\left[ {\begin{array}{*{20}l} {RNW_{a} \cap b} \hfill & {RN_{a} \cap b} \hfill & {RNE_{a} \cap b} \hfill \\ {RW_{a} \cap b} \hfill & {RB_{a} \cap b} \hfill & {RE_{a} \cap b} \hfill \\ {RSW_{a} \cap b} \hfill & {RS_{a} \cap b} \hfill & {RSE_{a} \cap b} \hfill \\ \end{array} } \right]\left[ {\begin{array}{*{20}l} {DNW_{a} \cap b} \hfill & {DN_{a} \cap b} \hfill & {DNE_{a} \cap b} \hfill \\ {DW_{a} \cap b} \hfill & {DB_{a} \cap b} \hfill & {DE_{a} \cap b} \hfill \\ {DSW_{a} \cap b} \hfill & {DS_{a} \cap b} \hfill & {DSE_{a} \cap b} \hfill \\ \end{array} } \right]} \right]$$

In this paper, for convenience, if such intersection is empty, it is recorded by 0, else recorded by 1 in such matrix. For instance, as shown in Fig. 1, we have *c UNE*:*UE a*. We can also use the direction relation matrix *P* to represent the direction of c with respect to a. That is to say, the direction matrix *P* is the equivalent representation of the cardinal direction relation *UNE*:*UE*.

2 $$P = \left[ {\left[ {\begin{array}{*{20}c} 0 & 0 & 1 \\ 0 & 0 & 1 \\ 0 & 0 & 1 \\ \end{array} } \right]\;\left[ {\begin{array}{*{20}c} 0 & 0 & 0 \\ 0 & 0 & 0 \\ 0 & 0 & 0 \\ \end{array} } \right]\;\left[ {\begin{array}{*{20}c} 0 & 0 & 0 \\ 0 & 0 & 0 \\ 0 & 0 & 0 \\ \end{array} } \right]} \right]$$

### Definition 5

Let *R* ∈ *TD*. The inverse of relation *R*, denoted by *INV*(*R*), is another 3D cardinal direction relation which satisfies the following. For any objects *a*,* b* ∈ *Tbox*, *a R b* holds, iff *b INV*(*R*)* a* holds.

### Definition 6

A basic 3D cardinal direction relation *R* is called *rectangular* iff there exist two cubes (with sides parallel to the *x*-axes, *y*-axes and *z*-axes) *a* and *b* such that *a R b* is satisfied; otherwise it is called non-rectangular. The set of these relations is denoted by *TD*_*rec*_.

Interval Algebra was introduced by Allen for temporal reasoning. There are thirteen basic relations between two temporal intervals which forms a set of jointly exhaustive and pairwise disjoint relations. The set of symbols *A*_*int*_ = {*p*, *m*, *o*, *s*, *d*, *f*, *pi*, *mi*, *oi*, *si*, *di*, *fi*, *eq*} is used to denote the thirteen basic relations.

In similar way, Balbiani introduced *n*-dimensional block algebra which is the *n*-dimensional extension of the interval algebra. For every integer *n* $\ge$ 1, a basic *n*-dimensional block algebra relation is an *n*-tuple like (*p*_1_, *p*_2_, …, *p*_*n*_) where *p*_*i*_ $\in${*p*, *m*, *d*, *s*, *f*, *e*, *mi*, *bi*, *si*, *oi*, *fi*, o}, for every *i* $\in${1, 2, …, *n*}, and for arbitrary *n*–blocks *a* and *b*, *a* and *b* satisfy the algebra relation (*p*_1_, *p*_2_, …, *p*_*n*_) if and only if the two intervals *a*_*i*_ and *b*_*i*_ satisfy the interval algebra relation* p*_*i*_ where *a*_*i*_ and *b*_*i*_ are the projection of *a* and *b* onto the *i-*th axis respectively, for every *i* $\in${1, 2, …, *n*}.

### Example 2

A basic 3-dimensional algebraic relation is denoted by (*k*_1_, *k*_2_, *k*_3_) where *k*_*i*_ $\in${*p*, *m*, *d*, *s*, *f*, *e*, *mi*, *bi*, *si*, *oi*, *di*, *fi*, o} and *i* $\in${1, 2, 3}.

**Figure 2:**
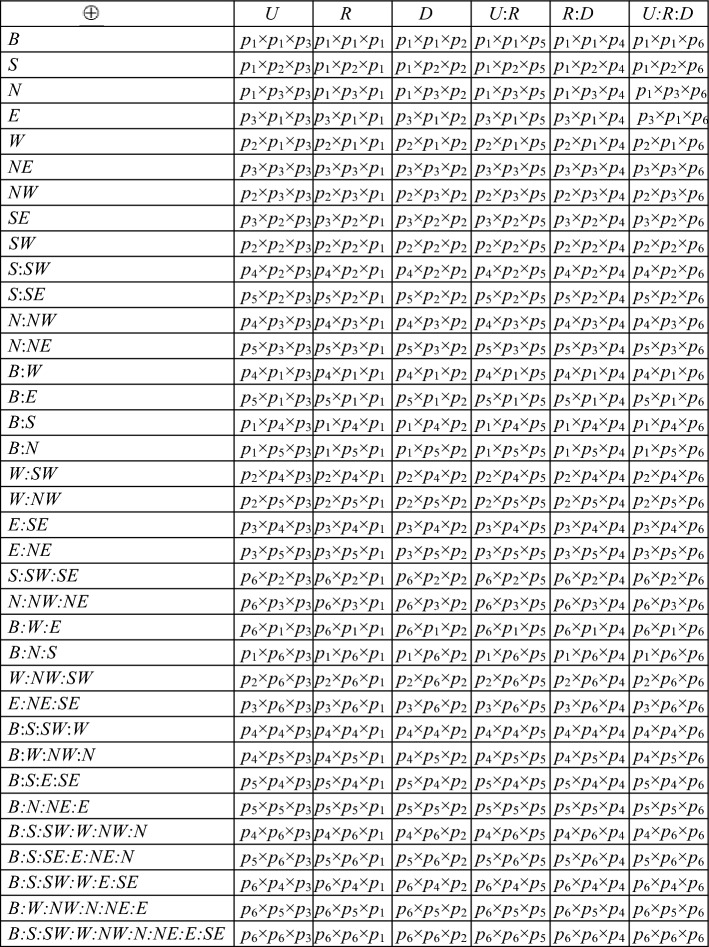
The mapping between the 3D rectangular cardinal direction relations and 3D interval relation matrix.

By the definition of the basic 3D cardinal direction relations and of the 3-block Algebra, there exists a 3-block algebra relation *r* such that a *R b* holds iff a *r b* holds for any basic 3D rectangular cardinal direction *R*, that is to say, there exists a mapping between basic 3D rectangular cardinal direction relations and 3-block Algebra relations which presented in our work^33^. In Fig. 2, the Algebra relation is presented by a Cartesian product (×) of the set of the interval relations *k*_1_ = {*d*, *s*, *f*, *e*},* k*_2_ = {*m*, *b*},* k*_3_ = {*mi*, *b*i},* k*_4_ = {*fi*, *o*}, *k*_5_ = {*si*, *oi*} and *k*_6_ = {*di*} and the basic 3D rectangular cardinal direction relation is presented by means of an operator $\oplus$ which is defined by the formulation.

3 $$r_{1:} r_{2:} \ldots_{:} r_{j} \oplus z_{1:} \ldots z_{k} = r_{1:} z_{1} r_{2:} \ldots_{:} z_{1} r_{j:} \ldots z_{k} r_{1:} z_{k} r_{2:} \ldots_{:} z_{k} r_{j}$$

where: ${r}_{1}, \dots ,{r}_{j}\in \{\text{N},\text{NE},\text{E},\text{SE},\text{B},\text{S},\text{SW},\text{W},\text{NW}\}$, ${z}_{1},\dots ,{z}_{k}\in \{\text{U},\text{R},\text{D}\}$ and 1 ≤ j ≤ 9, 1 ≤ k ≤ 3. For example S:SW:W ⊕ R:D = RS:RSW:RW:DS:DSW:DW.

## 3D direction relation matrix

In this paper, we convert the set of interval relations (*k*_1_ = {*d*, *s*, *f*, *e*},* k*_2_ = {*m*, *b*},* k*_3_ = {*mi*, *b*i},* k*_4_ = {*fi*, *o*}, *k*_5_ = {*si*, *oi*} and *k*_6_ = {*di*}) into matrix form to obtain the interval relation matrices([0 1 0], [1 0 0], [0 0 1], [1 1 0], [0 1 1], [1 1 1]). The set of interval relation matrices is denoted by (*p*_1_,* p*_2_,* p*_3_,* p p*_4_,* p*_5_,* p*_6_). In similar way, we convert the results of the inverse operation of the interval relations into the form of matrix as Table 1.

**Table 1:** The mapping of interval algebra.

Symbolic	Interval algebra	Interval matrix	Inverse interval matrix
*p*_1_	{*d*, *s*, *f*, e}	[0 1 0]	[0 1 0] [0 1 1] [1 1 1] [1 1 0]
*p*_2_	*{m*, b}	[1 0 0]	[0 0 1]
*p*_3_	{mi, bi}	[0 0 1]	[1 0 0]
*p*_4_	*{fi*, o}	[1 1 0]	[0 1 1] [0 1 0]
*p*_5_	{*si*, *oi*}	[0 1 1]	[0 1 0] [1 1 0]
*p*_6_	{di}	[1 1 1]	[0 1 0]

In order to fully use the advantages of the matrix, we build the mapping between the 3D interval relation matrix and basic rectangular cardinal direction relations which is presented in the form of matrix (see Fig. 2) where *p*_1_ = [0 1 0],* p*_2_ = [1 0 0],* p*_3_ = [0 0 1],* p*_4_ = [1 1 0],* p*_5_ = [0 1 1] and *p*_6_ = [1 1 1]. In Fig. 2, the 3D interval relations matrix is presented by a Cartesian product (×) of the set of the interval relations matrix *p*_1_ = [0 1 0],* p*_2_ = [1 0 0],* p*_3_ = [0 0 1],* p*_4_ = [1 1 0],* p*_5_ = [0 1 1] and *p*_6_ = [1 1 1] and the basic 3D rectangular cardinal direction relation is presented by means of an operator $\oplus$ which is defined by the formulation 1.

### Example 3

For the 3D rectangle cardinal direction relations *UB*:*UN:UNE*:*UE*, its corresponding 3D interval relations matrix is (*p*_5_,* p*_5_,* p*_3_) = ([0 1 1], [0 1 1], [0 0 1]).

For any integer *n* ≥ 1, the set of basic* n*-dimensional interval relation matrices have 13^*n*^ elements, denoted *B*_*n*_. The *n*-dimensional spatial object *a* satisfies the interval relation matrix (*p*_1_,* p*_2_, …, *p*_*n*_) with respect to *b*, denoted *a* (*p*_1_, *p*_2_, …, *p*_*n*_)* b*. Then, using the *n*-dimensional interval relation matrices of *B*_*n*_ as our basis, we can define the set *A*_*n*_ which is the power set $2^{{B_{n} }}$ of *B*_*n*_ and contains $2^{{13^{n} }}$ relations.

**Figure 3:**
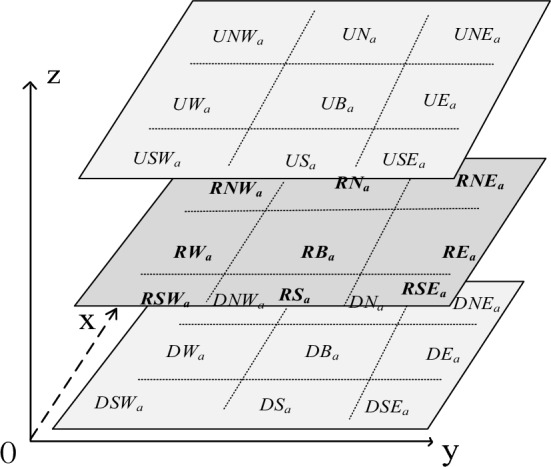
3D direction relation matrix.

We further build that the mapping between the basic 3D rectangular cardinal direction relations and the 3D direction relations matrix (see Fig. 3), on the basis of the above definition and the mapping of the 3D rectangular cardinal direction relations and the 3D interval relations matrix.

### Example 4

For *a DS b*, we have *a* ([0 1 0], [1 0 0], [1 0 0]) *b*. And for *a* ([0 1 0], [1 0 0], [1 0 0]) *b*, we have the following holds.

4 $$a \, \left[ {\left. {\left[ {\begin{array}{*{20}c} 0 & 0 & 0 \\ 0 & 0 & 0 \\ 0 & 0 & 0 \\ \end{array} } \right.} \right]\left[ {\begin{array}{*{20}c} 0 & 0 & 0 \\ 0 & 0 & 0 \\ 0 & 0 & 0 \\ \end{array} } \right]\left[ {\begin{array}{*{20}c} 0 & 0 & 0 \\ 0 & 0 & 0 \\ 0 & 1 & 0 \\ \end{array} } \right]} \right] \, b$$

### Definition 7

A basic cardinal direction relation matrix *P* is called rectangular direction relation matrix if all the non-zero elements in* P* form a rectangle; otherwise it is called non-rectangular direction relation matrix.

### Definition 8

Let *P* $\in$ *A*_*n*_, the inverse operation of an *n*-dimensional interval relation matrix *P* is denoted as *P*^−1^ and satisfies that for any *n*-dimensional spatial objects *a*, *b*, if *a P*^−1^* b* holds, if and only if *b P a* holds.

### Theorem 1

If the *n*-dimensional interval relation matrix (*p*_1_, *p*_2_, …, *p*_*n*_)$\in$ *B*_*n*_, then we have (*p*_1_,* p*_2_,…, *p*_*n*_)^−1^ = (*p*_1_^–1^,* p*_2_^–1^, …, *p*_*n*_^−1^) and if the *n*-dimensional interval relation matrix *P* $\in$ *A*_*n*_, then we have *P*^−1^ = {*p*^−1^$\left| p \right. \in$ *P*}.

### Proof

For any *n-*dimensional spatial objects *a* and *b*, *a*_*i*_ and *b*_*i*_ are the projections of *a* and *b* on the *i-th* dimension, respectively, where *i* $\in$ {1, 2, …, n} and *a* (*p*_1_,* p*_2_, …, *p*_*n*_)* b*. According to the definition of *n*-dimensional interval relation matrix, there exist *i* $\in$ {1, 2, …, *n*} such that *a*_*i*_* p*_*i*_* b*_*i*_. According to Definition 6, there exist* b*_*i*_* p*_*i*_^−1^ *a*_*i*_ such that *b* (*p*_1_^–1^,* p*_2_^–1^, …, *p*_*n*_^−1^) *a* where *i* $\in${1, 2, …, *n*}. and thus (*p*_1_,* p*_2_,…, *p*_*n*_)^−1^ = (*p*_1_^–1^,* p*_2_^–1^, …, *p*_*n*_^−1^). In similar way, for the *n*-dimensional interval relation matrix *P* $\in$ *A*_*n*_, we have *P*^−1^ = {*p*^−1^$\left| p \right. \in$ *P*}. And thus the theorem holds.

## Computing the inverse of 3D cardinal direction relations based on matrices

In this work, we proposed an algorithm for computing the inverse of 3D cardinal direction relations based on direction matrix, and the following definitions and theorems will be offered before this algorithm is presented.

### Definition 9

Let *P*, *Q* be two basic cardinal direction matrices. For each *i*, *j*, *k* ∈ {1, 2, 3}, if *Q*_*ijk*_ = 1, there must have *P*_*ijk*_ = 1, then we say that *P* includes *Q*.

### Example 5

$$\left[ {\left. {\left[ {\begin{array}{*{20}c} 0 & 1 & 1 \\ 0 & 1 & 1 \\ 0 & 1 & 1 \\ \end{array} } \right.} \right]\left[ {\begin{array}{*{20}c} 0 & 0 & 0 \\ 0 & 0 & 0 \\ 0 & 0 & 0 \\ \end{array} } \right]\left[ {\begin{array}{*{20}c} 0 & 0 & 0 \\ 0 & 0 & 0 \\ 0 & 0 & 0 \\ \end{array} } \right]} \right]$$ includes $$\left[ {\left. {\left[ {\begin{array}{*{20}c} 0 & 1 & 1 \\ 0 & 1 & 1 \\ 0 & 1 & 0 \\ \end{array} } \right.} \right]\left[ {\begin{array}{*{20}c} 0 & 0 & 0 \\ 0 & 0 & 0 \\ 0 & 0 & 0 \\ \end{array} } \right]\left[ {\begin{array}{*{20}c} 0 & 0 & 0 \\ 0 & 0 & 0 \\ 0 & 0 & 0 \\ \end{array} } \right]} \right]$$, and $$\left[ {\left. {\left[ {\begin{array}{*{20}c} 0 & 1 & 1 \\ 0 & 1 & 1 \\ 0 & 1 & 0 \\ \end{array} } \right.} \right]\left[ {\begin{array}{*{20}c} 0 & 0 & 0 \\ 0 & 0 & 0 \\ 0 & 0 & 0 \\ \end{array} } \right]\left[ {\begin{array}{*{20}c} 0 & 0 & 0 \\ 0 & 0 & 0 \\ 0 & 0 & 0 \\ \end{array} } \right]} \right]$$ is the original matrix of $$\left[ {\left. {\left[ {\begin{array}{*{20}c} 0 & 1 & 1 \\ 0 & 1 & 1 \\ 0 & 1 & 1 \\ \end{array} } \right.} \right]\left[ {\begin{array}{*{20}c} 0 & 0 & 0 \\ 0 & 0 & 0 \\ 0 & 0 & 0 \\ \end{array} } \right]\left[ {\begin{array}{*{20}c} 0 & 0 & 0 \\ 0 & 0 & 0 \\ 0 & 0 & 0 \\ \end{array} } \right]} \right]$$.

### Definition 10

Let *P* be a basic direction matrix. The bounding matrix of *P*, denoted by *Br*(*R*) is the smallest rectangular direction matrix (with respect to the number of non-zero elements) that includes *P*.

### Example 6

$$Br\left( {\left[ {\left. {\left[ {\begin{array}{*{20}c} 0 & 1 & 1 \\ 0 & 1 & 1 \\ 0 & 1 & 0 \\ \end{array} } \right.} \right]\left[ {\begin{array}{*{20}c} 0 & 1 & 1 \\ 0 & 1 & 1 \\ 0 & 1 & 0 \\ \end{array} } \right]\left[ {\begin{array}{*{20}c} 0 & 0 & 0 \\ 0 & 0 & 0 \\ 0 & 0 & 0 \\ \end{array} } \right]} \right]} \right) = \left[ {\left. {\left[ {\begin{array}{*{20}c} 0 & 1 & 1 \\ 0 & 1 & 1 \\ 0 & 1 & 1 \\ \end{array} } \right.} \right]\left[ {\begin{array}{*{20}c} 0 & 1 & 1 \\ 0 & 1 & 1 \\ 0 & 1 & 1 \\ \end{array} } \right]\left[ {\begin{array}{*{20}c} 0 & 0 & 0 \\ 0 & 0 & 0 \\ 0 & 0 & 0 \\ \end{array} } \right]} \right]$$

### Definition 11

Let *P* be a rectangular direction matrix, the original matrix of *P* is a basic direction matrix with that its bounding matrix is *P*. The set of original matrices of *P* is denoted by *ORG*(*P*).

### Example 7

Let P be $$\left[ {\left[ {\begin{array}{*{20}c} 0 & 0 & 0 \\ 0 & 0 & 0 \\ 0 & 0 & 0 \\ \end{array} } \right]\left[ {\begin{array}{*{20}c} 0 & 1 & 1 \\ 0 & 1 & 1 \\ 0 & 0 & 0 \\ \end{array} } \right]\left[ {\begin{array}{*{20}c} 0 & 0 & 0 \\ 0 & 0 & 0 \\ 0 & 0 & 0 \\ \end{array} } \right]} \right]$$. There are fifteen matrices included by P. There are five matrices included by *P*, as following.

$$\begin{aligned} ORG(P) & = \left( {\left[ {\left[ {\begin{array}{*{20}c} 0 & 0 & 0 \\ 0 & 0 & 0 \\ 0 & 0 & 0 \\ \end{array} } \right]\left[ {\begin{array}{*{20}c} 0 & 1 & 1 \\ 0 & 1 & 1 \\ 0 & 0 & 0 \\ \end{array} } \right]\left[ {\begin{array}{*{20}c} 0 & 0 & 0 \\ 0 & 0 & 0 \\ 0 & 0 & 0 \\ \end{array} } \right]} \right],\left[ {\left[ {\begin{array}{*{20}c} 0 & 0 & 0 \\ 0 & 0 & 0 \\ 0 & 0 & 0 \\ \end{array} } \right]\left[ {\begin{array}{*{20}c} 0 & 1 & 1 \\ 0 & 1 & 0 \\ 0 & 0 & 0 \\ \end{array} } \right]\left[ {\begin{array}{*{20}c} 0 & 0 & 0 \\ 0 & 0 & 0 \\ 0 & 0 & 0 \\ \end{array} } \right]} \right],} \right. \\ & \quad \left[ {\left[ {\begin{array}{*{20}c} 0 & 0 & 0 \\ 0 & 0 & 0 \\ 0 & 0 & 0 \\ \end{array} } \right]\left[ {\begin{array}{*{20}c} 0 & 1 & 1 \\ 0 & 0 & 1 \\ 0 & 0 & 0 \\ \end{array} } \right]\left[ {\begin{array}{*{20}c} 0 & 0 & 0 \\ 0 & 0 & 0 \\ 0 & 0 & 0 \\ \end{array} } \right]} \right],\left[ {\left[ {\begin{array}{*{20}c} 0 & 0 & 0 \\ 0 & 0 & 0 \\ 0 & 0 & 0 \\ \end{array} } \right]\left[ {\begin{array}{*{20}c} 0 & 0 & 1 \\ 0 & 1 & 1 \\ 0 & 0 & 0 \\ \end{array} } \right]\left[ {\begin{array}{*{20}c} 0 & 0 & 0 \\ 0 & 0 & 0 \\ 0 & 0 & 0 \\ \end{array} } \right]} \right], \\ & \quad \left. {\left[ {\left[ {\begin{array}{*{20}c} 0 & 0 & 0 \\ 0 & 0 & 0 \\ 0 & 0 & 0 \\ \end{array} } \right]\left[ {\begin{array}{*{20}c} 0 & 1 & 0 \\ 0 & 1 & 1 \\ 0 & 0 & 0 \\ \end{array} } \right]\left[ {\begin{array}{*{20}c} 0 & 0 & 0 \\ 0 & 0 & 0 \\ 0 & 0 & 0 \\ \end{array} } \right]} \right]} \right) \\ \end{aligned}$$

Before the algorithm for computing the inverse of 3D cardinal direction relations based on matrix is presented, we focus on the problem of solving the original relation for basic 3D rectangular cardinal direction relations. Firstly we use the 3 × 3 × 3 integer array and the depth-first search algorithm to find a solution that satisfies the condition, and those solutions are counted and outputted. This algorithm is presented as follows.

**Algorithm:**
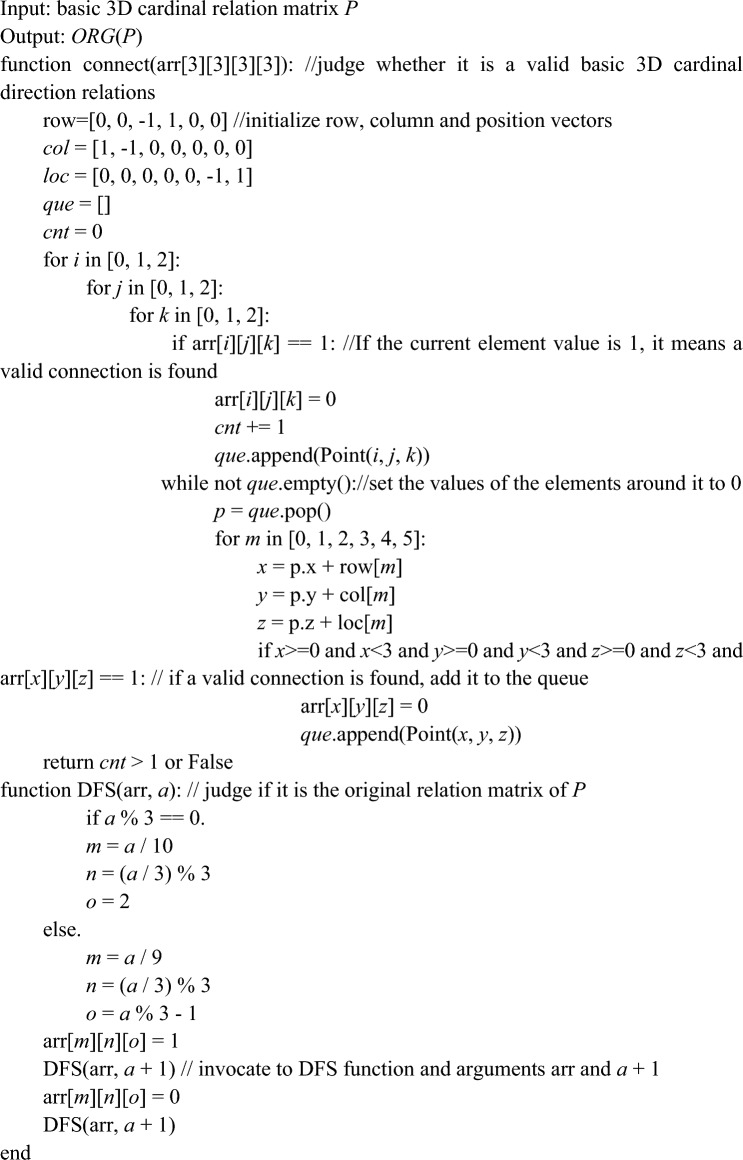
COMPUTE_ORG_3D ( ).

### Theorem 2

The original relation reasoning algorithm COMPUTE_ORG_3D( ) for the basic 3D cardinal direction relations is correct and complete.

### Proof

For any 3D direction relations matrix *P*, in order to verify whether its corresponding basic 3D cardinal direction relations satisfies the conditions of Definition 2, the algorithm first defines a CONNECT function. Then, use a nested for loop to iterate through all the points of the 3D array arr[ ] and determine whether it constitutes a connected graph. If *P* is a valid basic 3D cardinal direction relations, the DFS function is started to be executed to determine whether the 3D array satisfies the conditions of Definition 9. The functions and for loops of the algorithm realize the results of Definition 2 and Definition 9. Therefore, the algorithm COMPUTE_ORG_3D ( ) is correct and complete. And thus the theorem holds.

### Lemma 1

Let *a*, *b* $\in$ *Tbox*, if *b P a* and *P* $\in$ *TD*, so that *mbb*(*b*) *Br*(*P*)* a*.

### Lemma 2

Let* P* $\in$ *TD*, there exists $P^{ - 1} = \mathop \cup \limits_{{t \in (Br(P))^{ - 1} }} ORG(t)$.

### Theorem 3

Let* P* $\in$ *TD*_*rec*_, (*R*, *S*, *T*) is an interval relation matrix equivalent to *R*, where (*R*, *S*, *T*)$\in${[0 1 0], [1 0 0], [0 0 1], [1 1 0], [0 1 1], [1 1 1]}. And for any 3D interval relation matrix *p*, *RCD*(*p*) is a 3D rectangle cardinal direction relations equivalent to *p*. The inverse operation of *P is* computed as Eq. (5).

5 $$P^{ - 1} = \{ RCD(p)\left| {p \in (R^{ - 1} ,S^{ - 1} ,T^{ - 1} )} \right.\}$$

### Proof

$\forall m \in P^{ - 1}$, $\exists a,b \in T_{box}$, such that *a p b* and *b P a* holds, and there exists an interval relation matrix (*r*, *s*, *t*) such that *a*(*r*, *s*, *t*)*b* holds. According to Theorem 1, b (*r*^−1^,* s*^−1^,* t*^−1^) *a* holds, and (*R*, *S*, *T*) is an interval relation matrix equivalent to *P*, so (*r*^−1^,* s*^−1^,* t*^−1^)$\in$ (*R*, *S*, *T*). Therefore, we will get *r*^−1^ $\in$ R, *s*^−1^ $\in$ S, *t*^−1^ $\in$ T. According to Theorem 1, we get *r*^−1^ $\in$ *R*, *s*^−1^ $\in$ *S*, *t*^−1^ $\in$ *T* and (*r*, *s*, *t*) $\in$(*R*^−1^, *S*^−1^, *T*^−1^). So *RCD*((*r*, *s*, *t*))$\in${ *RCD*(*p*) $\left| p \right. \in$ (*R*^−1^,* S*^−1^,* T*^−1^)}, which is *m* $\in${*RCD*(*p*) $\left| p \right. \in$ (*R*^−1^,* S*^−1^,* T*^−1^)}. and thus $\forall m \in P^{ - 1}$, *m* $\in${*RCD*(*p*) $\left| p \right. \in$ (*R*^−1^,* S*^−1^,* T*^−1^)}.

Conversely, $\forall$ *m* $\in${*RCD*(*p*) $\left| p \right. \in$ (*R*^−1^,* S*^−1^,* T*^−1^)}, there exists $a,b \in T_{box}$ such that *a m b* holds. And there exists an interval operation matrix (*r*, *s*, *t*) $\in$ (*R*^−1^,* S*^−1^,* T*^−1^) such that *a* (*r*, *s*, *t*) *b* holds. According to Theorem 1 we have *b* (*r*^−1^,* s*^−1^,* t*^−1^) *a*. According to (*r*, *s*, *t*) $\in$ (*R*^−1^,* S*^−1^,* T*^−1^), we have (*r*^−1^,* s*^−1^,* t*^−1^) $\in$ (*R*, *S*, *T*). Since (*R*, *S*, *T*) is an interval operation matrix equivalent to *P*, *b* (*r*^−1^,* s*^−1^,* t*^−1^) *a* holds, then *b P a* holds. If *a p b* and *b P a*, according to Definition 6, m $\in$ *P*^−1^. Therefore,$\forall$ *m* $\in$ {*RCD*(*p*) $\left| p \right. \in$ (*R*^−1^,* S*^−1^,* T*^−1^)}, *m* $\in$ *P*^−1^ holds, and thus the theorem holds.

### Theorem 4

Let* P* $\in$ *TD*, (*R*, *S*, *T*) is an interval relation matrix equivalent to *R*, where (*R*, *S*, *T*)$\in$ {[0 1 0], [1 0 0], [0 0 1], [1 1 0], [0 1 1], [1 1 1]}, and for any 3D interval relation matrix *p*, *RCD*(*p*) is a 3D rectangle cardinal direction relations equivalent to *p*. The inverse operation of *P* is computed as Eq. (6).

6 $$P^{ - 1} = \mathop \cup \limits_{{t \in \{ RCD(p)\left| {p \in (R^{ - 1} ,S^{ - 1} ,T^{ - 1} )} \right.\} }} ORG(t)$$

### Proof

According to Lemma 2 we have $P^{ - 1} = \mathop \cup \limits_{{t \in (Br(P))^{ - 1} }} ORG(t)$, since (*R*, *S*, *T*) is an interval relation matrix equivalent to *P*. Then, According to Theorem 3 then we have (*Br*(P))^−1^ = {*RCD*(*p*) $\left| p \right. \in$ (*R*^−1^, *S*^−1^, *T*^−1^)}, and thus the theorem holds.

According to Theorem 4 and the mapping between the 3D rectangular cardinal direction relations and 3D interval relation matrix, the algorithm of the inverse of 3D cardinal direction relations based on matrix is given.

**Algorithm:**
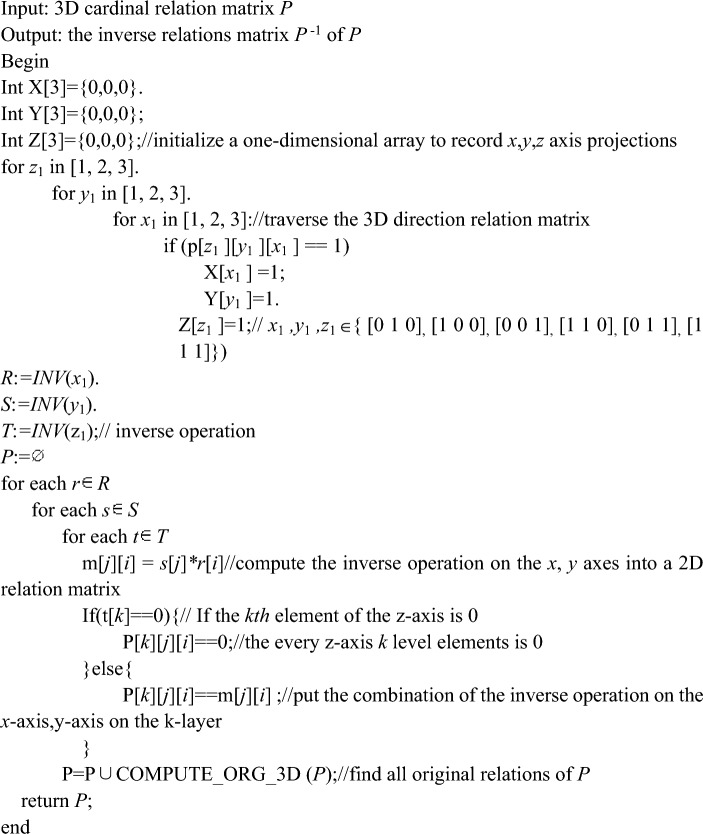
Compute_INV_3D(*P*)

### Theorem 5

The algorithm of the inverse of cardinal direction relations Compute_INV_3D( ) is correct and complete.

### Proof

For any 3D direction relations matrix *P*, firstly, project to *x-*axes, *y-*axes, *z-*axes to get the interval relation matrix {*x*_1_, *y*_1_, *z*_1_} which is equivalent to *P*. Then, the interval relation matrix {*x*_1_, *y*_1_, *z*_1_} is inverted according to Definition 3. And after executing the *INV*( ) operation, we will get *P*^−1^ = {*RCD*(*p*) $\left| p \right. \in$ (*R*, *S*, *T*)}, where *R* = *INV*(*x*_1_), *S* = *INV*(*y*_1_), *and T* = *INV*(z_1_), and a proof of the correctness of this procedure has been verified in Theorem 3. Executing the final for loop gives $P^{ - 1} = \mathop \cup \limits_{{t \in \{ RCD(p)\left| {p \in (x_{1}^{ - 1} ,y_{1}^{ - 1} ,z_{1}^{ - 1} )} \right.\} }} ORG(t)$, the correctness of which has been verified in Theorem 4. Multiple arrays, for loops and if statements in the algorithm realize the results of the operations of Finding, Theorem 3 and Theorem 4. Therefore, the algorithm Compute_INV_3D( ) is correct and complete, and thus the theorem holds.

## Verification results and discussions

In this section, We have implemented the Algorithm COMPUTE_ORG_3D ( ), Compute_INV_3D( ) in C programming language that runs on Visual Studio 2019. The main idea of this program is to record the projection of any 3D direction relations matrix to *x*, *y*, *z-*axes by initializing a one-dimensional array and traversing the 3D direction relations matrix through a for loop. We can conclude the 3D direction relations matrix *P* which is equal to the 3D direction relations matrix *P* interval relation matrix (*x*_1_, *y*_1_, *z*_1_). And it is inverted to combine the inverse operation on the *x*, *y-*axes into a 2D direction relations matrix. and then continue to traverse the elements of the z-axis to output the 3D direction relations matrix that satisfies the conditions. Finally, we can solve for all the original relations of each 3D direction relations matrix.

The inverse of each basic 3D direction matrix has been generated by algorithm Compute_INV_3D( ) and manual reasoning respectively. The results and the code are available from us. Then, we will check whether the result of Algorithm Compute_INV_3D( ) is consistent with that of manual reasoning for eachbasic 3D cardinal direction relation.

For instance, the inverse of cardinal direction relation UN:UNE:UE:RN:RNE:RE can by generated by means of the inverse operation of its corresponding direction matrix $$\left[ {\left. {\left[ {\begin{array}{*{20}c} 0 & 1 & 1 \\ 0 & 0 & 1 \\ 0 & 0 & 0 \\ \end{array} } \right.} \right]\left[ {\begin{array}{*{20}c} 0 & 1 & 1 \\ 0 & 0 & 1 \\ 0 & 0 & 0 \\ \end{array} } \right]\left[ {\begin{array}{*{20}c} 0 & 0 & 0 \\ 0 & 0 & 0 \\ 0 & 0 & 0 \\ \end{array} } \right]} \right]$$*.*

Firstly, we calculate the inverse of $$\left[ {\left. {\left[ {\begin{array}{*{20}c} 0 & 1 & 1 \\ 0 & 0 & 1 \\ 0 & 0 & 0 \\ \end{array} } \right.} \right]\left[ {\begin{array}{*{20}c} 0 & 1 & 1 \\ 0 & 0 & 1 \\ 0 & 0 & 0 \\ \end{array} } \right]\left[ {\begin{array}{*{20}c} 0 & 0 & 0 \\ 0 & 0 & 0 \\ 0 & 0 & 0 \\ \end{array} } \right]} \right]$$ by using the algorithm Compute_INV_3D( ) for computing the inverse of basic 3D cardinal direction relations. We have the matrix to the *x-*axes, *y-*axes, *z-*axes respectively to get its equivalent interval relation matrix {[0 1 1], [0 1 1], [0 1 1]}. According to Theorem 3, We have that:

$$\begin{aligned} & INV\left( {\left[ {\left. {\left[ {\begin{array}{*{20}c} 0 & 1 & 1 \\ 0 & 0 & 1 \\ 0 & 0 & 0 \\ \end{array} } \right.} \right]\left[ {\begin{array}{*{20}c} 0 & 1 & 1 \\ 0 & 0 & 1 \\ 0 & 0 & 0 \\ \end{array} } \right]\left[ {\begin{array}{*{20}c} 0 & 0 & 0 \\ 0 & 0 & 0 \\ 0 & 0 & 0 \\ \end{array} } \right]} \right]} \right) \\ & = \{ RCD(p)\left| {p \in (\left[ {0 \, 1 \, 1} \right]^{ - 1} ,\left[ {0 \, 1 \, 1} \right]^{ - 1} ,\left[ {0 \, 1 \, 1} \right]^{ - 1} )} \right.\} \\ & { = }\{ RCD(p)\left| {p \in ((\left[ {0 \, 1 \, 0} \right],\left[ {1 \, 1 \, 0} \right]),(\left[ {0 \, 1 \, 0} \right],\left[ {1 \, 1 \, 0} \right]),(\left[ {0 \, 1 \, 0} \right],\left[ {1 \, 1 \, 0} \right]))} \right.\} \\ & { = }\{ RCD(p)\left| {p \in ((\left[ {0 \, 1 \, 0} \right],\left[ {0 \, 1 \, 0} \right],\left[ {0 \, 1 \, 0} \right]),(\left[ {0 \, 1 \, 0} \right],\left[ {0 \, 1 \, 0} \right],\left[ {1 \, 1 \, 0} \right]),} \right.(\left[ {0 \, 1 \, 0} \right],\left[ {1 \, 1 \, 0} \right],\left[ {0 \, 1 \, 0} \right]), \\ & \quad \quad \quad \quad \quad \quad \;(\left[ {0 \, 1 \, 0} \right],\left[ {1 \, 1 \, 0} \right],\left[ {1 \, 1 \, 0} \right]),(\left[ {1 \, 1 \, 0} \right],\left[ {0 \, 1 \, 0} \right],\left[ {0 \, 1 \, 0} \right]),(\left[ {1 \, 1 \, 0} \right],\left[ {0 \, 1 \, 0} \right],\left[ {1 \, 1 \, 0} \right]), \\ & \quad \quad \quad \quad \quad \quad \;(\left[ {1 \, 1 \, 0} \right],\left[ {1 \, 1 \, 0} \right],\left[ {0 \, 1 \, 0} \right]), \, (\left[ {1 \, 1 \, 0} \right],\left[ {1 \, 1 \, 0} \right],\left[ {1 \, 1 \, 0} \right]))\} \\ \end{aligned}$$

Then, we convert those 8 interval relation matrices into 8 3D direction relation matrix by using the last if statement. And find all the original relations of each 3D direction relation matrix which is shown as follow.

$$\begin{aligned} & INV\left( {\left[ {\left. {\left[ {\begin{array}{*{20}c} 0 & 1 & 1 \\ 0 & 0 & 1 \\ 0 & 0 & 0 \\ \end{array} } \right.} \right]\left[ {\begin{array}{*{20}c} 0 & 1 & 1 \\ 0 & 0 & 1 \\ 0 & 0 & 0 \\ \end{array} } \right]\left[ {\begin{array}{*{20}c} 0 & 0 & 0 \\ 0 & 0 & 0 \\ 0 & 0 & 0 \\ \end{array} } \right]} \right]} \right) \\ & = ORG\left( {\left[ {\left. {\left[ {\begin{array}{*{20}c} 0 & 0 & 0 \\ 0 & 0 & 0 \\ 0 & 0 & 0 \\ \end{array} } \right.} \right]\left[ {\begin{array}{*{20}c} 0 & 0 & 0 \\ 0 & 1 & 0 \\ 0 & 0 & 0 \\ \end{array} } \right]\left[ {\begin{array}{*{20}c} 0 & 0 & 0 \\ 0 & 0 & 0 \\ 0 & 0 & 0 \\ \end{array} } \right]} \right]} \right) \, \cup \, ORG\left( {\left[ {\left. {\left[ {\begin{array}{*{20}c} 0 & 0 & 0 \\ 0 & 0 & 0 \\ 0 & 0 & 0 \\ \end{array} } \right.} \right]\left[ {\begin{array}{*{20}c} 0 & 0 & 0 \\ 0 & 1 & 0 \\ 0 & 0 & 0 \\ \end{array} } \right]\left[ {\begin{array}{*{20}c} 0 & 0 & 0 \\ 0 & 1 & 0 \\ 0 & 0 & 0 \\ \end{array} } \right]} \right]} \right) \\ & \quad \cup ORG\left( {\left[ {\left. {\left[ {\begin{array}{*{20}c} 0 & 0 & 0 \\ 0 & 0 & 0 \\ 0 & 0 & 0 \\ \end{array} } \right.} \right]\left[ {\begin{array}{*{20}c} 0 & 0 & 0 \\ 0 & 1 & 0 \\ 0 & 1 & 0 \\ \end{array} } \right]\left[ {\begin{array}{*{20}c} 0 & 0 & 0 \\ 0 & 0 & 0 \\ 0 & 0 & 0 \\ \end{array} } \right]} \right]} \right) \, \cup \, ORG\left( {\left[ {\left. {\left[ {\begin{array}{*{20}c} 0 & 0 & 0 \\ 0 & 0 & 0 \\ 0 & 0 & 0 \\ \end{array} } \right.} \right]\left[ {\begin{array}{*{20}c} 0 & 0 & 0 \\ 0 & 1 & 0 \\ 0 & 1 & 0 \\ \end{array} } \right]\left[ {\begin{array}{*{20}c} 0 & 0 & 0 \\ 0 & 1 & 0 \\ 0 & 1 & 0 \\ \end{array} } \right]} \right]} \right) \\ & \quad \cup ORG\left( {\left[ {\left. {\left[ {\begin{array}{*{20}c} 0 & 0 & 0 \\ 0 & 0 & 0 \\ 0 & 0 & 0 \\ \end{array} } \right.} \right]\left[ {\begin{array}{*{20}c} 0 & 0 & 0 \\ 1 & 1 & 0 \\ 0 & 0 & 0 \\ \end{array} } \right]\left[ {\begin{array}{*{20}c} 0 & 0 & 0 \\ 0 & 0 & 0 \\ 0 & 0 & 0 \\ \end{array} } \right]} \right]} \right) \, \cup \, ORG\left( {\left[ {\left. {\left[ {\begin{array}{*{20}c} 0 & 0 & 0 \\ 0 & 0 & 0 \\ 0 & 0 & 0 \\ \end{array} } \right.} \right]\left[ {\begin{array}{*{20}c} 0 & 0 & 0 \\ 1 & 1 & 0 \\ 0 & 0 & 0 \\ \end{array} } \right]\left[ {\begin{array}{*{20}c} 0 & 0 & 0 \\ 1 & 1 & 0 \\ 0 & 0 & 0 \\ \end{array} } \right]} \right]} \right) \\ & \quad \cup ORG\left( {\left[ {\left. {\left[ {\begin{array}{*{20}c} 0 & 0 & 0 \\ 0 & 0 & 0 \\ 0 & 0 & 0 \\ \end{array} } \right.} \right]\left[ {\begin{array}{*{20}c} 0 & 0 & 0 \\ 1 & 1 & 0 \\ 1 & 1 & 0 \\ \end{array} } \right]\left[ {\begin{array}{*{20}c} 0 & 0 & 0 \\ 0 & 0 & 0 \\ 0 & 0 & 0 \\ \end{array} } \right]} \right]} \right) \, \cup \, ORG\left( {\left[ {\left. {\left[ {\begin{array}{*{20}c} 0 & 0 & 0 \\ 0 & 0 & 0 \\ 0 & 0 & 0 \\ \end{array} } \right.} \right]\left[ {\begin{array}{*{20}c} 0 & 0 & 0 \\ 1 & 1 & 0 \\ 1 & 1 & 0 \\ \end{array} } \right]\left[ {\begin{array}{*{20}c} 0 & 0 & 0 \\ 1 & 1 & 0 \\ 1 & 1 & 0 \\ \end{array} } \right]} \right]} \right) \\ \end{aligned}$$

**Figure 4:**
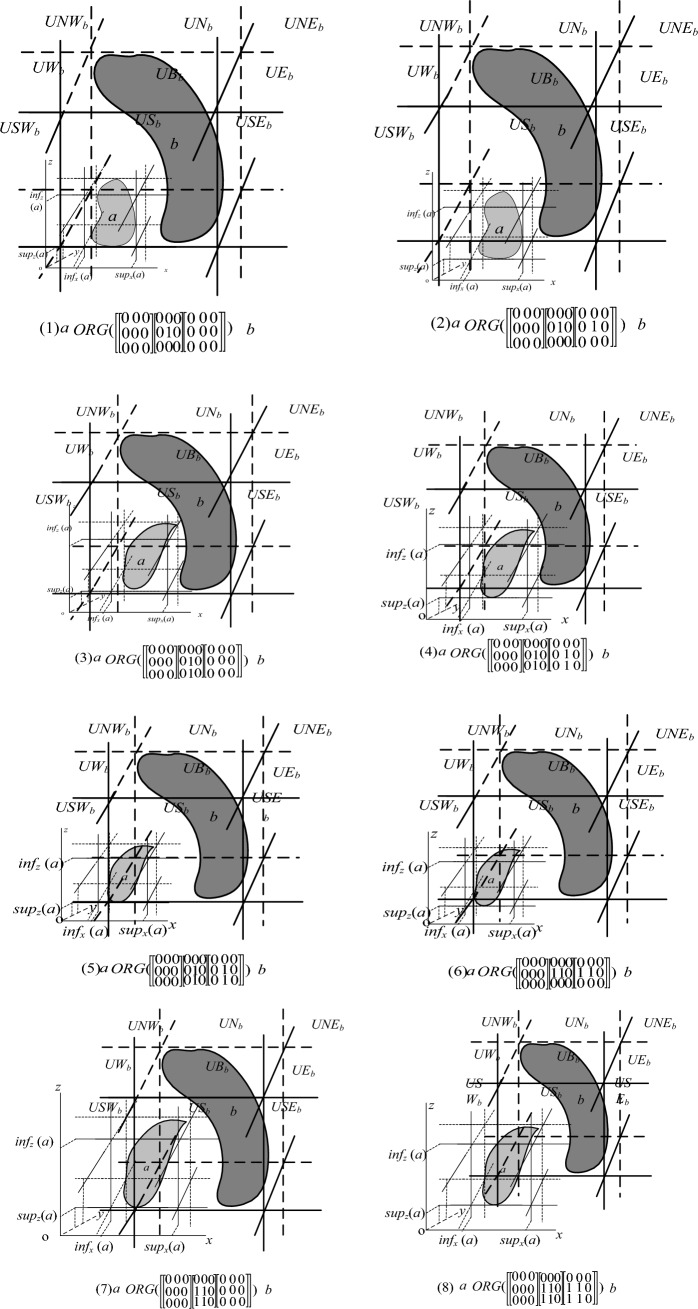
spatial configuration.

In fact, for *a*, *b* $\in$ *Tbox*, we know that *b* $$\left[ {\left. {\left[ {\begin{array}{*{20}c} 0 & 1 & 1 \\ 0 & 0 & 1 \\ 0 & 0 & 0 \\ \end{array} } \right.} \right]\left[ {\begin{array}{*{20}c} 0 & 1 & 1 \\ 0 & 0 & 1 \\ 0 & 0 & 0 \\ \end{array} } \right]\left[ {\begin{array}{*{20}c} 0 & 0 & 0 \\ 0 & 0 & 0 \\ 0 & 0 & 0 \\ \end{array} } \right]} \right]$$ *a*, and all the possible spatial configuration of *a* and *b* are shown in Fig. 4, the detailed results are as follows:

$$\begin{aligned} & a \, ORG\left( {\left[ {\left. {\left[ {\begin{array}{*{20}c} 0 & 0 & 0 \\ 0 & 0 & 0 \\ 0 & 0 & 0 \\ \end{array} } \right.} \right]\left[ {\begin{array}{*{20}c} 0 & 0 & 0 \\ 0 & 1 & 0 \\ 0 & 0 & 0 \\ \end{array} } \right]\left[ {\begin{array}{*{20}c} 0 & 0 & 0 \\ 0 & 0 & 0 \\ 0 & 0 & 0 \\ \end{array} } \right]} \right]} \right)b,a \, ORG\left( {\left[ {\left. {\left[ {\begin{array}{*{20}c} 0 & 0 & 0 \\ 0 & 0 & 0 \\ 0 & 0 & 0 \\ \end{array} } \right.} \right]\left[ {\begin{array}{*{20}c} 0 & 0 & 0 \\ 0 & 1 & 0 \\ 0 & 0 & 0 \\ \end{array} } \right]\left[ {\begin{array}{*{20}c} 0 & 0 & 0 \\ 0 & 1 & 0 \\ 0 & 0 & 0 \\ \end{array} } \right]} \right]} \right)b, \\ & a \, ORG\left( {\left[ {\left. {\left[ {\begin{array}{*{20}c} 0 & 0 & 0 \\ 0 & 0 & 0 \\ 0 & 0 & 0 \\ \end{array} } \right.} \right]\left[ {\begin{array}{*{20}c} 0 & 0 & 0 \\ 0 & 1 & 0 \\ 0 & 1 & 0 \\ \end{array} } \right]\left[ {\begin{array}{*{20}c} 0 & 0 & 0 \\ 0 & 0 & 0 \\ 0 & 0 & 0 \\ \end{array} } \right]} \right]} \right)b,a \, ORG\left( {\left[ {\left. {\left[ {\begin{array}{*{20}c} 0 & 0 & 0 \\ 0 & 0 & 0 \\ 0 & 0 & 0 \\ \end{array} } \right.} \right]\left[ {\begin{array}{*{20}c} 0 & 0 & 0 \\ 0 & 1 & 0 \\ 0 & 1 & 0 \\ \end{array} } \right]\left[ {\begin{array}{*{20}c} 0 & 0 & 0 \\ 0 & 1 & 0 \\ 0 & 1 & 0 \\ \end{array} } \right]} \right]} \right)b, \\ \end{aligned}$$

$$\begin{aligned} & a \, ORG\left( {\left[ {\left. {\left[ {\begin{array}{*{20}c} 0 & 0 & 0 \\ 0 & 0 & 0 \\ 0 & 0 & 0 \\ \end{array} } \right.} \right]\left[ {\begin{array}{*{20}c} 0 & 0 & 0 \\ 1 & 1 & 0 \\ 0 & 0 & 0 \\ \end{array} } \right]\left[ {\begin{array}{*{20}c} 0 & 0 & 0 \\ 0 & 0 & 0 \\ 0 & 0 & 0 \\ \end{array} } \right]} \right]} \right)b,a \, ORG\left( {\left[ {\left. {\left[ {\begin{array}{*{20}c} 0 & 0 & 0 \\ 0 & 0 & 0 \\ 0 & 0 & 0 \\ \end{array} } \right.} \right]\left[ {\begin{array}{*{20}c} 0 & 0 & 0 \\ 1 & 1 & 0 \\ 0 & 0 & 0 \\ \end{array} } \right]\left[ {\begin{array}{*{20}c} 0 & 0 & 0 \\ 1 & 1 & 0 \\ 0 & 0 & 0 \\ \end{array} } \right]} \right]} \right)b, \\ & a \, ORG\left( {\left[ {\left. {\left[ {\begin{array}{*{20}c} 0 & 0 & 0 \\ 0 & 0 & 0 \\ 0 & 0 & 0 \\ \end{array} } \right.} \right]\left[ {\begin{array}{*{20}c} 0 & 0 & 0 \\ 1 & 1 & 0 \\ 1 & 1 & 0 \\ \end{array} } \right]\left[ {\begin{array}{*{20}c} 0 & 0 & 0 \\ 0 & 0 & 0 \\ 0 & 0 & 0 \\ \end{array} } \right]} \right]} \right)b,a \, ORG\left( {\left[ {\left. {\left[ {\begin{array}{*{20}c} 0 & 0 & 0 \\ 0 & 0 & 0 \\ 0 & 0 & 0 \\ \end{array} } \right.} \right]\left[ {\begin{array}{*{20}c} 0 & 0 & 0 \\ 1 & 1 & 0 \\ 1 & 1 & 0 \\ \end{array} } \right]\left[ {\begin{array}{*{20}c} 0 & 0 & 0 \\ 1 & 1 & 0 \\ 1 & 1 & 0 \\ \end{array} } \right]} \right]} \right)b. \\ \end{aligned}$$

Form Fig. 4, we have that the result of our algorithm is consistent with the actual situation for the direction matrix $$\left[ {\left. {\left[ {\begin{array}{*{20}c} 0 & 1 & 1 \\ 0 & 0 & 1 \\ 0 & 0 & 0 \\ \end{array} } \right.} \right]\left[ {\begin{array}{*{20}c} 0 & 1 & 1 \\ 0 & 0 & 1 \\ 0 & 0 & 0 \\ \end{array} } \right]\left[ {\begin{array}{*{20}c} 0 & 0 & 0 \\ 0 & 0 & 0 \\ 0 & 0 & 0 \\ \end{array} } \right]} \right]$$. Therefore our algorithm can work correctly for reasoning with the inverse of its corresponding cardinal direction relation UN:UNE:UE:RN:RNE:RE. Furthermore, we compare the result of our algorithm Compute_INV_3D( ) with the actual situation by manual reasoning shown as Fig. 4 for each of the other basic cardinal direction relations defined by 3DR27 model, respectively. We find that the result of our algorithm is consistent with the actual situation for any basic cardinal direction relation. Therefore our algorithm is correct and complete.

Obviously, the manual reasoning is a very complicated operation and requires a lot of work. The proposed algorithm Compute_INV_3D( ) can calculate the inverse of any basic cardinal direction relation directly which does not need to use reference tables and any manual operation. Our work completely realized the automatic inference and calculation of the inverse of the basic cardinal direction relations in the 3DR27 model.

## Conclusion

In this paper, we use a 3 × 3 × 3 direction relation matrix to describe and define the direction relations between spatial objects. By using the equivalent connection between the 3D rectangular cardinal direction relations and 3D interval relation matrix, we focus on the inversion operation for 3D cardinal direction relations defined by the 3DR27 model. We developed an algorithm for computing the inverse of cardinal direction relations by means of 3D cardinal direction relations matrix. In this study, we solved the problem of the automatically computing the inverse operation of the 3D cardinal direction relations by means of the excellent properties of matrix operations.

Notice that our algorithm does not need any manual reasoning and calculation, and the help of the reference tables, which enhances the ability of intelligent reasoning of 3DR27 model. In this paper, the inverse of 3D cardinal direction relations is studied based on the matrix, and the consistency test of the basic 3D cardinal direction relations network will be investigated by using the interval relation matrix in the future research.

## Data Availability

All data generated or analysed during this study are included in this published article.
